# Influence of Axial Load and a 45-Degree Flexion Head Position on Cervical Spinal Stiffness in Healthy Young Adults

**DOI:** 10.3389/fphys.2021.786625

**Published:** 2021-12-23

**Authors:** Léonie Hofstetter, Melanie Häusler, Petra Schweinhardt, Ursula Heggli, Denis Bron, Jaap Swanenburg

**Affiliations:** ^1^Integrative Spinal Research ISR, Department of Chiropractic Medicine, Balgrist University Hospital, Zurich, Switzerland; ^2^Faculty of Medicine, University of Zurich, Zurich, Switzerland; ^3^AeMC, Aeromedical Center, Swiss Air Forces, Dubendorf, Switzerland

**Keywords:** cervical spine, flexion, axial load, stiffness, posture

## Abstract

**Background:** Neck pain is a major cause of disability worldwide. Poor neck posture such as using a smartphone or work-related additional cervical axial load, such headgear of aviators, can cause neck pain. This study aimed at investigating the role of head posture or additional axial load on spinal stiffness, a proxy measure to assess cervical motor control.

**Methods:** The posterior-to-anterior cervical spinal stiffness of 49 young healthy male military employees [mean (SD) age 20 ± 1 years] was measured in two head positions: neutral and 45-degree flexed head position and two loading conditions: with and without additional 3 kg axial load. Each test condition comprised three trials. Measurements were taken at three cervical locations, i.e., spinous processes C2 and C7 and mid-cervical (MC).

**Results:** Cervical spinal stiffness measurements showed good reliability in all test conditions. There was a significant three-way interaction between location × head position × load [*F*(2, 576) = 9.305, *p* < 0.001]. Significant two-way interactions were found between measurement locations × loading [*F*(2, 576) = 15.688, *p* < 0.001] and measurement locations × head position [*F*(2, 576) = 9.263, *p* < 0.001]. There was no significant interaction between loading × head position [*F*(1, 576) = 0.692, *p* = 0.406]. *Post hoc* analysis showed reduction of stiffness in all three measurement locations in flexion position. There was a decrease in stiffness in C2 with loading, increase in stiffness in C7 and no change in MC.

**Discussion:** A flexed head posture leading to decreased stiffness of the cervical spine might contribute to neck pain, especially if the posture is prolonged and static, such as is the case with smartphone users. Regarding the additional load, stiffness decreased high cervical and increased low cervical. There was no change mid cervical. The lower spinal stiffness at the high cervical spine might be caused by capsular ligament laxity due to the buckling effect. At the lower cervical spine, the buckling effect seems to be less dominant, because the proximity to the ribs and sternum provide additional stiffness.

## Introduction

Neck pain is a common problem and one of the highest contributors to disability worldwide (James et al., 2018). An awkward head position is the most commonly reported physical risk factor for a first episode of neck pain (Kim et al., 2018), and working with the neck flexed at more than 20° has been suggested to increase this risk (Ariens et al., 2001). Awkward posture is exacerbated when using a smartphone (Lee et al., 2015), which is associated with strain of cervical extensor muscles, altered postural control and pain (Eitivipart et al., 2018). In addition to awkward head position, cervical axial load can contribute to neck pain. For example, neck pain can occur in people who endure increased cervical axial load, such as aviators wearing headgear and individuals carrying loads with their heads (Echarri and Forriol, 2002; Geere et al., 2010; Posch et al., 2019). In sports such as gymnastics, ice hockey, American football, and rugby, which have a high incidence of neck injury, axial loading of the cervical spine is considered the primary mechanism of injury (Torg et al., 2002; Barile et al., 2007; Trewartha et al., 2015).

To protect the neck from pain and injury due to flexion position and/or axial load, a functional cervical motor control system is needed (Posch et al., 2019). Motor control, which consists of active, passive and neurological subsystems (Panjabi, 1992), can be accessed via different proxy measures. Most commonly, muscle activity, i.e., the active subsystem, is measured (Stokes and Gardner-Morse, 2003; Honkanen et al., 2017). The passive subsystem has also been studied, mainly *in vitro* using human spines or porcine models, typically reduced to bones and ligaments (Gardner-Morse and Stokes, 2003; Stokes and Gardner-Morse, 2003; Zhang et al., 2020). The assessment of spinal stiffness *in vivo* is considered a proxy measure of the active and passive subsystems combined (Swanenburg et al., 2018, 2020; Hausler et al., 2020).

It has been observed recently that lumbar and thoracic spinal stiffness is dependent on body position and axial load (Hausler et al., 2020; Swanenburg et al., 2020). Thoracic and lumbar spinal stiffness was found to increase while standing, compared with a prone position (Hausler et al., 2020). With increased axial load, either added via adding an additional axial load larger than 45% of the body weight with the help of a long weight bar or during hypergravity induced by parabolic flight, spinal stiffness decreased (Swanenburg et al., 2018, 2020; Hausler et al., 2020; Glaus et al., 2021).

The relationships between axial load, head flexion position and cervical spinal stiffness are yet to be determined, despite increasing evidence showing a relationship between neck pain and cervical flexion. Therefore, this study aimed to investigate the effects of cervical flexion position, with or without additional cervical axial load, on cervical spinal stiffness.

## Materials and Methods

A total of 49 healthy young adult male participants were recruited, aged 18–23 years and employees of the Swiss military. Swiss military personnel were selected because they are used to wearing a helmet. Written informed consent was obtained from all participants. The exclusion criteria were: any current or chronic neck pain, age younger than 18 years and a Neck Disability Index (NDI) score of more than 15 points. The NDI is a self-report questionnaire with 10 items assessing: pain intensity, personal care, lifting, work, headaches, concentration, sleeping, driving, reading and recreation. Each item is scored on a 0–5 scale. Zero means no disability, 5 complete disability. The scores are summed, resulting in a total score between 0 and 50. The NDI German Version has demonstrated good reliability (Swanenburg et al., 2014). The study was approved by the Ethics Committee of the Canton of Zurich (Reference: BASEC 2019–00830) (ClinicalTrials.gov Identifier: NCT04434235).

### Data Collection Procedures

First, demographic data, such as sex, age, weight, and height of each participant were collected. After completing the NDI questionnaire, participants were asked to sit with a straight back on a workout bench. Two of the three measurement locations [the spinous processes of C2 (high-cervical) and C7 (low-cervical)] were manually identified by two experienced manual therapists, with the spinous process of C2 being the most cranial one that can clearly be palpated and using the flexion-extension test to locate C7 (Povoa et al., 2018). C2 and C7 were marked with ink to label the location for spinal stiffness assessment and the marking was verified by both therapists to increase accuracy. For the third mid-cervical (MC) assessment location, half of the distance between the spinous process of C2 and C7 was taken.

#### Head Position

**FIGURE 1:**
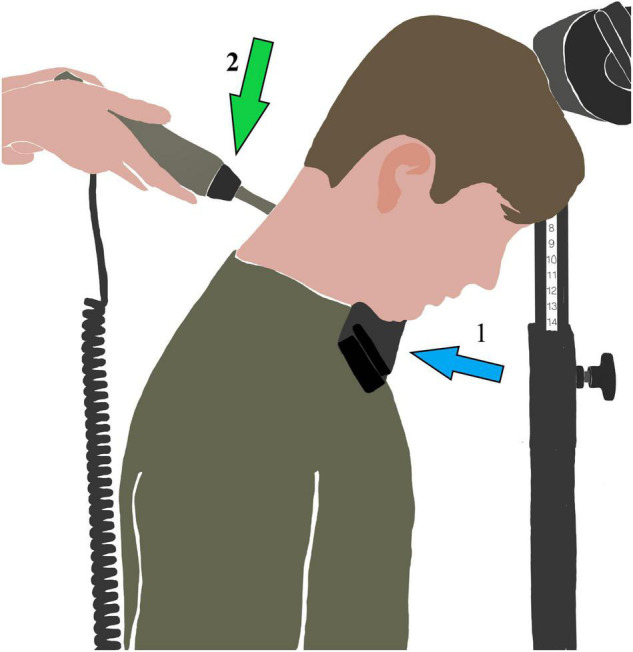
Experimental set-up in cervical flexion position. Arrow 1: Size-adjustable foam pad. Arrow 2: Stiffness device.

The first spinal stiffness measurements were conducted sitting straight with their hands placed relaxed on their thighs and with a neutral head position, with the forehead lightly touching against a horizontal bar, to guarantee position. The common head flexion angle while using a smartphone is 45 degrees from vertical (Lee et al., 2015; Guan et al., 2016). Therefore, the flexion condition was performed with 45-degree flexion, by asking the participant to put a size-adjustable foam pad between the sternum and the chin to guarantee constant head position (Figure 1). The size of the foam pad was defined before the measurements. First, the 45° flexion head position was determined using an electronic goniometer (EasyAngle^®^, Meloq AB, Stockholm, Sweden). Then, a size-adjustable foam pad was placed between the sternum and the chin to keep the angle. Pre-testing showed no effect of the foam pad on the stiffness results.

#### Load

**FIGURE 2:**
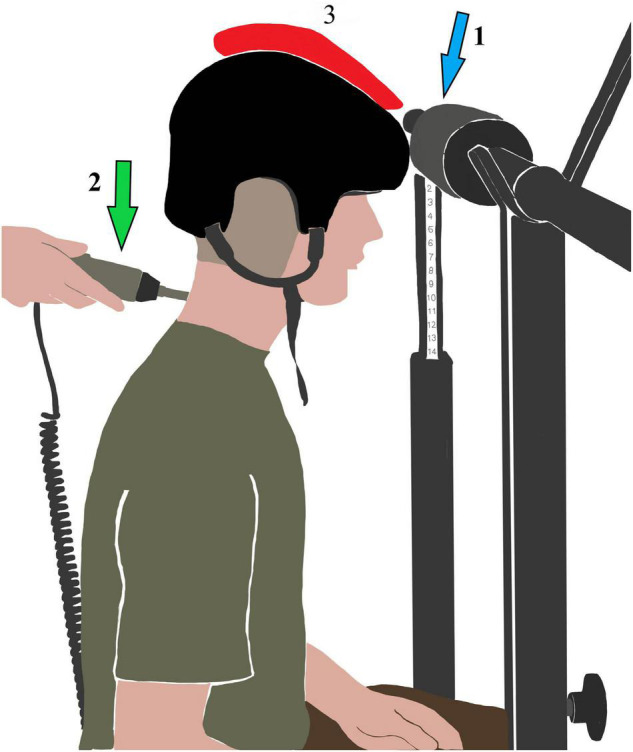
Experimental set-up in neutral position with additional axial load (red). Arrow 1: Foam pad. Arrow 2: Stiffness device.

After the neutral and flexion measurements, the participants were asked to put on an ice hockey helmet (size M) (CCM, Saint Laurent, QC, Canada) to recreate the real world-working situation. Additional load was fixed to the helmet, on the sagittal balance axis, resulting in a total helmet weight of 3 kg (Figure 2). This weight was chosen because helmets of helicopter pilots weigh up to 2.5 kg (Lange et al., 2013). Then, measurements in both head positions were repeated with additional axial load. For each test situation, the measurements were repeated three times. The participants were asked to inform the examiner if they experienced any pain during measurement.

### Cervical Spinal Stiffness Assessments

A computer-assisted device (PulStarFRAS, Sense Technology Inc., Pittsburgh, PA) was used to measure posterior-to-anterior spinal stiffness (Leach et al., 2003). This device possesses good test–retest reliability, with intraclass correlation coefficient (ICC) values greater than 0.83 (Hausler et al., 2020). Spinal stiffness is defined as the impulse response to the deformation of the spine system; a linear, time-invariant response to a very short (<1 ms) impulse. This time invariance allows the impulse response to be measure in Newton, rather than Newton seconds (as in classical measurements) (Girod et al., 2003). A preload of 18 N was applied to trigger the assessment and overcome possible confounders caused by soft tissue components. After reaching the preload of 18 Newton, the device automatically applied an impulse with a single contact probe—a force of 27 N in a 90° angle relative to the surface of the back.

### Statistical Analysis

Baseline characteristics of study participants were summarized using descriptive statistics. Mean cervical spinal stiffness and 95% confidence intervals (CI) of each measurement location in all testing situations were plotted graphically.

#### Reliability

ICC with 95% CI was calculated to assess test–retest reliability. To determine absolute reliability and standard error of measurement (SEM) were calculated. Cronbach’s alpha was calculated to evaluate internal consistency.

#### Influence of Measurement Locations, Head Position, and Axial Load

A three-way ANOVA was used to determine if there are interaction effects between the three independent variables head position (neutral and flexion), loading (unloaded and loaded), and measurement locations (C2, MC, and C7) regarding the dependent variable cervical stiffness. An alpha level of 0.05 was used to determine statistical significance. *Post hoc* tests were calculated to investigate the factors head position, loading. All statistical analyses were performed using SPSS 23 (IBM, Chicago, IL).

## Results

### Participants

**FIGURE 3:**
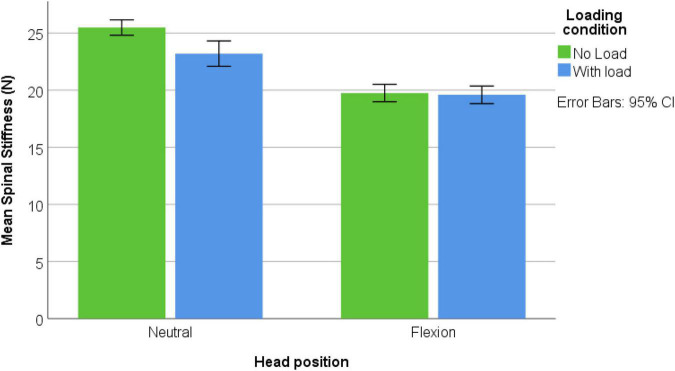
C2 location; spinal stiffness mean values in both head positions and both loading conditions.

**FIGURE 4:**
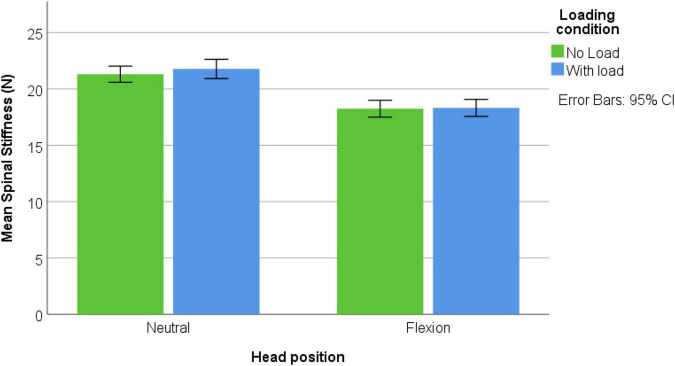
MC location; spinal stiffness mean values in both head positions and both loading conditions.

**FIGURE 5:**
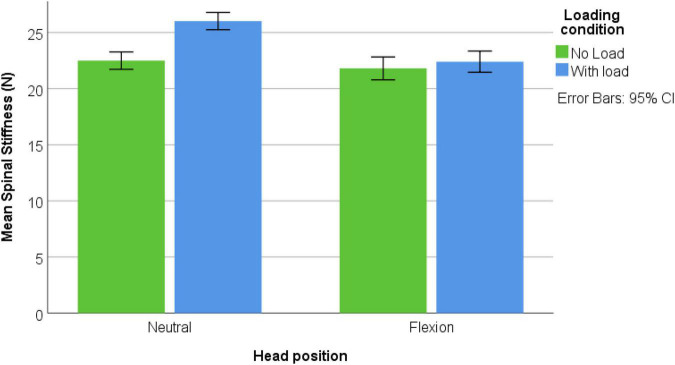
C7 location; spinal stiffness mean values in both head positions and both loading conditions.

Forty-nine male participants were recruited [mean age: 19.9 ± (SD) 1.1 years; mean height: 179.8 ± 11.4 cm; mean weight: 74.4 ± 11.4 kg]. None were excluded and none reported pain during the assessments. Figures 3–5 represents mean spinal stiffness with 95% CI for all three measurement locations, in both head positions and loading conditions.

### Reliability

Spinal stiffness measurements showed good reliability in both head position and measurement locations with ICCs > 0.799 and Cronbach’s alpha 0.808—0.948. All reliability values of both head position, two loading conditions, and measurement locations are shown in Table 1. All cervical stiffness values split in measurement locations are shown in Figures 3–5.

**TABLE 1:** Reliability of the three stiffness measurements in all four test situations at al all three locations.

	C2			MC			C7		
	ICC (95% CI)	A	SEM (N)	ICC (95% CI)	A	SEM (N)	ICC (95% CI)	A	SEM (N)
Neutral	0.875 (0.799–0.925)	0.874	1.016	0.799(0.677–0.880)	0.883	1.534	0.810(0.695–0.887)	0.808	1.537
Flexion	0.873(0.796–0.924)	0.878	1.226	0.868(0.811–0.930)	0.866	0.931	0.883(0.813–0.930)	0.883	1.267
Neutral + loaded	0.872(0.795–0.924)	0.872	1.056	0.818(0.708–0.892)	0.815	1.401	0.844(0.749–0.907)	0.843	1.528
Flexion + loaded	0.891 (0.824–0.935)	0.889	0.870	0.948(0.946–0.969)	0.948	0.430	0.916(0.866–0.950)	0.916	0.840

*ICC, intraclass correlation coefficient; CI, confidence interval; A, Cronbach’s alpha; N, Newton; SEM, standard error of measurement; Neutral + loaded, neutral head position with additional axial load; Flexion + loaded, flexed head position with additional axial load; MC, mid-cervical: C2 and C7, cervical vertebra 2 and 7.*

### Influence of Measurement Locations, Head Position, and Axial Load

Levene’s test showed that the assumption of homogeneity of variance was satisfied. A three-way ANOVA was performed to examine the interaction effects of head position, loading, and measurement location regarding cervical spinal stiffness. There was a significant three-way interaction [*F*(2, 576) = 9.305, *p* < 0.001]. Significant two-way interactions were found between loading x measurement location [*F*(2, 576) = 15.688, *p* < 0.001] and head position × measurement location [*F*(2, 576) = 9.263, *p* < 0.001]. There was no significant interaction between loading × head position [*F*(1, 576) = 0.692, *p* = 0.406]. *Post hoc* analysis showed reduction of stiffness in all three measurement locations in flexion position. There was a decrease in stiffness in C2 with loading, increase in stiffness in C7 and no change in MC. The *post hoc* results are shown in Table 2 and Supplementary Figures 1–3 show the respective interactions.

**TABLE 2:** *Post hoc* results for head position and loading conditions in all measurement locations.

	Cervical spinal stiffness (Newton)		
Measurement location	Neutral mean (SD)	Flexion mean (SD)	*p*
C2	24.35 (3.84)	19.67 (2.64)	<0.001*
MC	21.54 (2.45)	18.29 (2.60)	<0.001*
C7	24.26 (3.20)	22.10 (3.42)	<0.001*

	**Unloaded** **mean (SD)**	**Loaded** **mean (SD)**	***P***

C2	22.62 (3.80)	21.40 (3.77)	0.025*
MC	19.78 (2.97)	20.05 (3.29)	0.546
C7	22.15 (3.14)	24.21 (3.50)	<0.001*

*p < 0.05.

*MC, mid-cervical: C2 and C7, cervical vertebra 2 and 7.*

## Discussion

In this study, cervical spinal stiffness was examined in two different head positions, with and without additional axial load, at three cervical measurement locations. The measurements of spinal stiffness in asymptomatic individuals were found to be reliable. Our results show a reduction of cervical spinal stiffness in the 45-degree flexion position compared to the neutral position in all measurement locations.

An earlier study measured the effect of different head positions on cervical spinal stiffness in a prone body posture, not reporting any significant change between neutral and flexed position (Snodgrass and Rhodes, 2012). In contrast to our study, the stiffness (insignificantly) increased from neutral to flexion position. This contrasting result might be explained by prone body posture in which the head is fully supported, possibly leading to an increase in the measured stiffness. Further, neck stiffness was found to be significantly decreased after 10 min static neck flexion, after returning into neutral position (Mousavi-Khatir et al., 2018). This suggests that the decrease in stiffness that was found in the present study during flexion might remain, even if the head has returned to a neutral position.

### Head Position

The decrease in spinal stiffness in the flexion head position could be due to the flexion relaxation phenomenon (FRP) of the cervical spine, which describes a myoelectric “silence” of the neck extensor muscles during cervical flexion (Simon et al., 2006). This can be explained by a transfer of the extension moment from the active to the passive structures of the spine with further flexion (Pialasse et al., 2009). The FRP has been observed only for the cervical erector spinae muscle, whereas the upper trapezius muscle showed no FRP response (Colloca and Hinrichs, 2005; Maroufi et al., 2013). Furthermore, cervical flexor muscles, as antagonists, are likely to be less activated in the flexion position (Maroufi et al., 2013). At the low-cervical spine, a smaller decrease in stiffness was found in the 45-degree flexion position compared to the neutral head position. More likely, the low-cervical spine is at the end of motion given that the cervicothoracic junction experiences less flexion than other segments of the cervical spine (Nightingale et al., 2007). Therefore, this could have caused a smaller decrease in spinal stiffness. Overall, it can be hypothesized that less support by a less active muscular subsystem led to decreased cervical stiffness in the flexion head position.

Another explanation for the decrease in cervical spinal stiffness could be the pressure on the cervical intervertebral discs during flexion. During the movement of the neck in the different directions, the loads on the cervical intervertebral discs increases resulting in an increase in cervical intervertebral disc pressure (Bayoglu et al., 2019). Another study reported a twofold increase in pressure in the cervical discs with flexion and a fourfold increase of the shear forces (Barrett et al., 2020). This additional load, generated by flexion, leads to capsular ligament laxity of the facet joints (Steilen et al., 2014), the so-called buckling effect (Nightingale et al., 1996). By reducing the passive stability, the buckling effect might have decreased the spinal stiffness in the present study.

### Additional Load

The interaction effect between loading and measurement location showed that loading had some effect. A decrease in stiffness in the high cervical region and an increase in the low-cervical spine was observed. The change in stiffness in the high cervical spine due to the additional load might be explained by capsular ligament laxity due to the buckling effect (Nightingale et al., 1996). Thus, the reduced tension on the passive structures might have led to a play between the structures that resulted in a reduction in spinal stiffness. The unchanged spinal stiffness in the mid-cervical vertebrae might be the results of opposite effects that cancel each other out, namely muscle activity and buckling. In contrast to the high-cervical spine, more low cervical muscle activity is needed to stabilize not only the head and the additional load, but also the high-cervical spine (Bergmark, 1989; Swanenburg et al., 2020; Glaus et al., 2021). This increased muscle activation would be expected to lead to an increase in spinal stiffness (Swanenburg et al., 2020). Thus, the unchanged stiffness of the mid-cervical spine might be the net effect of buckling effects and increased muscle activity. The stiffness at the low-cervical spine increased with additional load. This increase in spinal stiffness can be explained by an increase in muscle activity to stabilize not only the head and extra load, but also the decreased stiffness of the high-cervical spine. Moreover, mobility is maximal at the low-cervical spine (Penning, 1978). Compression of the cervical spine due to the additional load and the consequent relaxation of the stabilizing ligaments appears to be less dominant in the low-cervical spine. More muscle activity is needed to stabilize the cervical spine because of the lesser passive stability (Izzo et al., 2013). Additionally, the low-cervical spine is closer to structures that provide additional stability, such as the ribs and the sternum. It could be argued that the more mobile low-cervical spine needs more muscle activation to stabilize the cervical spine with the additional load, which led to increased stiffness at the low-cervical spine.

### Clinical Implication

There is relationship between a flexion neck posture and neck pain symptoms (Barrett et al., 2020), especially if this flexed posture is sustained for a long period of time, as in the case of excessive use of mobile devices (Ariens et al., 2001; Bayoglu et al., 2019). Avoiding prolonged static postures and a flexed head position greater than 30 degrees might help to prevent neck pain.

### Limitations

The generalizability of the present study’s results is unknown because only asymptomatic young male subjects were included. Additionally, other factors that might influence cervical spinal stiffness were not measured. For example, muscle activity was not directly assessed by electromyography (EMG). The activity of the deep neck flexors, such as the longus capitis and longus colli, cannot be measured with superficial EMG; this would require the use of a nasopharyngeal catheter, the application of which would have been invasive and impractical in the study environment (Robinson et al., 2009). Individual differences in muscle dimensions that stabilize the cervical spine were not considered. The individuals’ cervical range of motion in flexion direction was not assessed. Nevertheless, all participants were able to assume the 45-degree flexion position without any difficulty.

## Conclusion

A flexed head posture leads to a decrease in the stiffness of the cervical spine. The decreased stiffness might be due to increased pressure and shear forces on the cervical intervertebral discs during a 45-degree flexion. It is expected that such effects would be pronounced when the posture is prolonged and static, such as is the case with smartphone users. Regarding the additional load, stiffness decreased high cervical and increased low cervical. There was and no change in mid cervical. The lower spinal stiffness at the high cervical spine might be caused by capsular ligament laxity due to the buckling effect. At the lower cervical spine, the buckling effect seems to be less dominant, because the proximity to the ribs and sternum provide additional stiffness.

## Data Availability Statement

The raw data supporting the conclusions of this article will be made available by the authors, without undue reservation.

## Ethics Statement

The studies involving human participants were reviewed and approved by the Ethics Committee of the Canton of Zurich (Reference: BASEC 2019–00830). The patients/participants provided their written informed consent to participate in this study.

## Author Contributions

JS developed the research question and the design. LH, MH, and UH conducted the data acquisition. LH, MH, and JS carried out analysis and interpretation of the results. LH produced an early version of the manuscript. JS, DB, LH, MH, UH, and PS revised the manuscript to bring it to its current version. All authors contributed to the article and approved the submitted version.

## Conflict of Interest

The authors declare that the research was conducted in the absence of any commercial or financial relationships that could be construed as a potential conflict of interest.

## Publisher’s Note

All claims expressed in this article are solely those of the authors and do not necessarily represent those of their affiliated organizations, or those of the publisher, the editors and the reviewers. Any product that may be evaluated in this article, or claim that may be made by its manufacturer, is not guaranteed or endorsed by the publisher.

## Abbreviations

CI: Confidence intervals
EMG: Electromyography
FRP: Flexion relaxation phenomenon
ICC: Intraclass correlation coefficient
NDI: Neck Disability Index
SEM: Standard error of measurement.
