# Supplementary material for: Influence of Axial Load and a 45-Degree Flexion Head Position on Cervical Spinal Stiffness in Healthy Young Adults
Source: Front Physiol. 2021 Dec 23;12:786625. doi: 10.3389/fphys.2021.786625 (PMC8733818; doi:10.3389/fphys.2021.786625)

Supplementary File 1

Figure S1. Interaction plot between head position and Loading condition
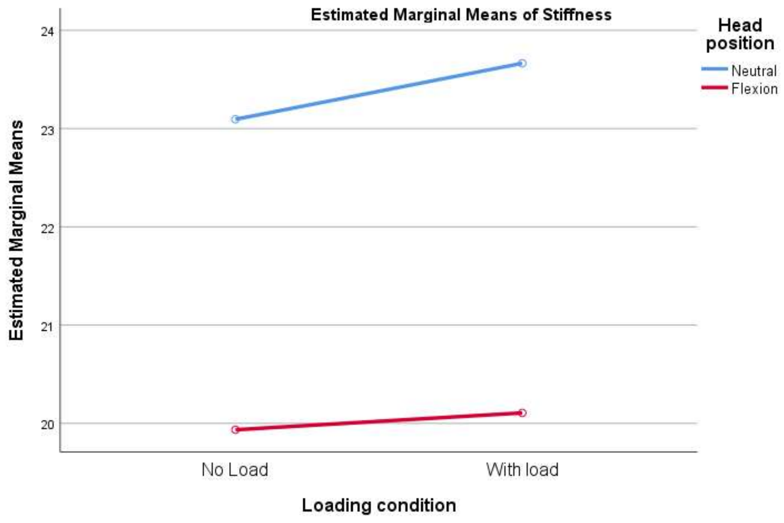
Figure S2 Interaction plot between head position and measurement location
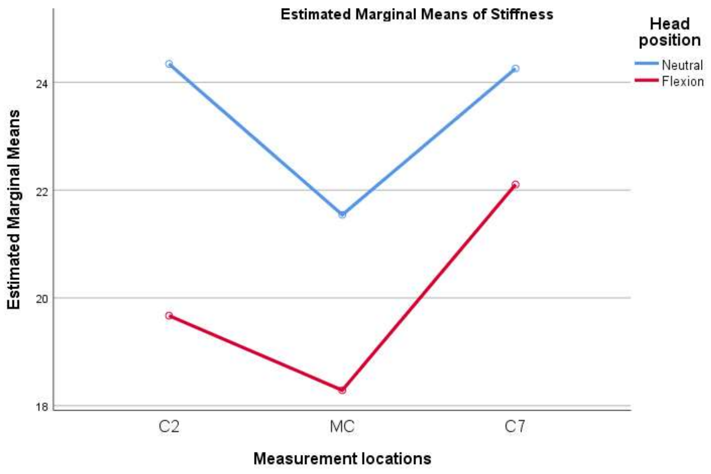

Figure S3 Interaction plot between loading condition and measurement location

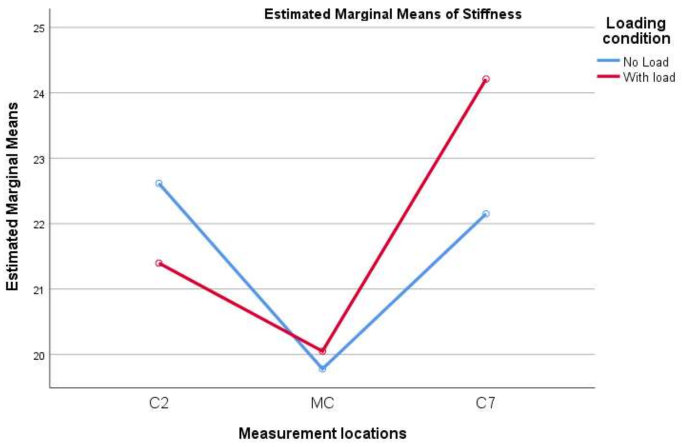

Supplement: Supplementary file 1 [file Data_Sheet_1.PDF]
